# *Taenia solium* cysticercosis in West Africa: status update

**DOI:** 10.1051/parasite/2018048

**Published:** 2018-09-18

**Authors:** Jihen Melki, Eugène Koffi, Marcel Boka, André Touré, Man-Koumba Soumahoro, Ronan Jambou

**Affiliations:** 1 Institut Pasteur de Côte d’Ivoire B.P. 490 Abidjan 01 Côte d’Ivoire; 2 Direction des Services Vétérinaires, Ministère des Ressources Animales et Halieutiques B.P. V84 Abidjan 01 Côte d’Ivoire; 3 Université Alassane Ouattara, Ministère de l’Enseignement Supérieur et de la Recherche Scientifique B.P. V18 Bouaké 01 Côte d’Ivoire; 4 Institut Pasteur 25–28 Rue du Dr Roux, 75015 Paris France

**Keywords:** Cysticercosis, *Taenia solium*, West Africa, epilepsy, pig

## Abstract

Cysticercosis is caused by the larvae of the cestode *Taenia solium*. Few data are available on the prevalence of this disease in pigs and humans in West African countries. The aim of this study was to provide an overview of existing data concerning the spread of this parasitosis in the countries of the Economic Community of West African States (ECOWAS) on the basis of the literature published over the last five decades. Systematic searches for publications were carried out on PubMed and Google Scholar, as well as in certain regional and local journals. From a total of 501 articles initially retrieved concerning *T. solium* cysticercosis in West African countries, only 120 articles were relevant for this review and therefore finally retained. For pigs, only eight out of sixteen countries of the region have reported porcine cysticercosis. Post-mortem examination of carcasses at slaughterhouses, meat inspection at butcheries or tongue inspection in herds have been the main source of data, but may not entirely reflect actual parasite distribution. For humans, only five out of sixteen countries reported epidemiological data on neurocysticercosis. Most data referred to neurocysticercosis prevalence among epileptic patients or isolated clinical cases. Furthermore, existing data are often old. Overall, *T. solium* cysticercosis remains largely neglected in West Africa, and its prevalence appears not to be affected by any religion in particular. There is an urgent need to promote and implement health partnerships and programs on this disease in order to collect more data and identify sensitive populations in the countries of the ECOWAS area.

## Abstract (fr)

La cysticercose est causée par les larves du cestode *Taenia solium*. Peu de données sont disponibles sur la prévalence de cette maladie chez les porcs et les humains dans les pays d’Afrique de l’Ouest. Le but de cette étude est de fournir un aperçu des données existantes concernant la propagation de cette parasitose dans les pays de la Communauté économique des États de l’Afrique de l’Ouest (CEDEAO) sur la base de la littérature publiée au cours des cinq dernières décennies. Des recherches systématiques de publications ont été effectuées sur PubMed, Google Scholar, ainsi que sur certaines revues régionales et locales. Sur un total de 501 articles initialement récupérés et concernant la cysticercose à *T. solium* dans les pays d’Afrique de l’Ouest, seuls 120 articles étaient pertinents pour cet examen et ont donc finalement été retenus. Pour les porcs, seulement huit des seize pays de la région ont signalé une cysticercose porcine. L’examen post mortem des carcasses dans les abattoirs, l’inspection de la viande dans les boucheries ou l’inspection de la langue dans le troupeau ont été la principale source de données, mais peuvent ne pas refléter entièrement la répartition réelle des parasites. Pour l’homme, seuls cinq pays sur seize ont rapporté des données épidémiologiques sur la neurocysticercose. La plupart des données se référaient à la prévalence de la neurocysticercose chez les épileptiques ou les cas cliniques isolés. De plus, les données existantes sont souvent anciennes. Dans l’ensemble, la cysticercose à *T. solium* reste largement négligée en Afrique de l’Ouest et sa prévalence ne semble être affectée par aucune religion en particulier. Il est urgent de promouvoir et de mettre en œuvre des partenariats et des programmes de santé sur cette maladie afin de collecter davantage de données et d’identifier les populations sensibles dans les pays de la zone CEDEAO.

## Introduction

Cysticercosis is a parasitic infection caused by the metacestode larval stage (Cysticercus) of *Taenia solium* [51]. The life cycle of *T. solium* is complex and requires two mammalian hosts: pigs and humans are intermediate hosts, whereas only humans are definitive hosts. Humans develop taeniasis through the consumption of raw or undercooked pork containing tapeworm larvae. In animal cysticercosis, tapeworm eggs are ingested with water and food. In human cysticercosis, an additional mode of transmission of the disease is autoinfection through hand-to-mouth contact with hands contaminated by (human) infected feces [54, 105]. Once in the mammalian host, the eggs mature into oncospheres, cross the intestinal wall, enter the bloodstream, and reach the host tissues where the metacestodes evolve into cysticerci [109]. When larvae are located in the brain, the infection is called neurocysticercosis (NCC), a frequent cause of epilepsy or epileptic seizures [99]. Autoinfection is frequent in humans. In West Africa, while a high prevalence of cysticercosis in pigs and humans has occasionally been reported, there is a lack of a consistent and systematic approach in the study of the disease, which may result in a misestimation of its prevalence [123]. West Africa is approximately 6 million km^2^ in area, covering 20% of the African continent [122]. It includes 16 countries, namely: Mauritania, Cape Verde, Ghana, Côte d’Ivoire, Senegal, Benin, Togo, Mali, Guinea, Niger, Liberia, Burkina Faso, Nigeria, The Gambia, Guinea Bissau and Sierra Leone. The West African population is about 368 million inhabitants [40], 46% of whom are concentrated in urban centers and more than 27% in Nigeria’s urban centers only [40, 66]. Rapid urbanization due to natural population growth and rural-urban migration has resulted in proliferation of slum areas with high population densities (e.g. Abuja, Nigeria), poor sanitation, and very low-standard housing. Population movements from rural to urban areas, often coupled with the lack of an adequate water supply and sewage systems, have facilitated the transmission of parasitic infections [78, 100].

Each West African country has its own particular history with diverse ethnic groups and an array of different languages, cultures and customs [87, 113]. Political and social factors affect the resources of the population and, therefore, their health and hygiene conditions. Nigeria has the largest economy in the area on account of its natural and agricultural resources [40]. Ghana and Senegal have benefited from a certain level of stability, enabling their economies to grow and develop [24]. Conversely, countries like Côte d’Ivoire, Guinea Bissau, Liberia, and Sierra Leone were crippled, for decades, by violent conflicts and civil wars causing socio-economic disruption, which made it extremely difficult to conduct health surveys [47]. Some of the countries that experienced civil wars, e.g. Guinea, Liberia and Sierra Leone, were also heavily affected by the Ebola outbreak [117].

The West African area is traversed by the “African Transition Zone” and divided into two regions: (1) the northern region, extending up to the Sahara Desert, and (2) the southern region, extending down to the tropics [20]. This Transition Zone also serves as a transition line between culture and religion: while Christianity is dominant in the south, Islam appears to be more common in the north (e.g. Niger, Mali, Burkina Faso and Mauritania) [34, 88]. This may therefore explain, at least in part, the difference in data availability on cysticercosis between south and north.

Climate can also shape parasite transmission [50]. The *arid zone* includes northern parts of Senegal, parts of Mali, Burkina Faso and Niger. The *semi-arid zone* covers the southern parts of Senegambia, Mali, Burkina Faso, Niger, and upper parts of Guinea-Bissau, Guinea, Togo, Benin and Nigeria. The *sub-humid zone* includes Guinea-Bissau, upper parts of Guinea, the southernmost parts of Mali and Burkina Faso and the northern parts of Ghana, Côte d’Ivoire, Sierra Leone, Benin and the central parts of Nigeria. Vegetation zones run parallel to each other, from north to south, and are related to rainfall quantity [50].

The high level of heterogeneity of the area seems to influence the transmission of the disease and could therefore explain the irregular prevalence of cysticercosis throughout the region. This review tries to shape the pattern of the disease in this area in relation with this heterogeneity. In the meantime, it highlights the scarcity of data available on *T*. *solium* cysticercosis and supports the need for more studies.

## Methods

This review covered all the 16 West African countries. The data on cysticercosis in West Africa were collected from: (1) peer-reviewed articles on *T. solium* cysticercosis in Africa, (2) grey literature consisting of written materials such as theses and dissertations obtained from Google Scholar, and (3) published reports regarding *T. solium* posted by United Nations-related agencies such as WHO, FAO, or OIE, and the Centers for Disease Control and Prevention (CDC).

The following search strategy was applied: (1) in PubMed, using the Boolean operator AND, the terms “cysticercosis”, “*Taenia solium*” and “West Africa’’ (and each individual country name); (2) in Google Scholar (http://scholar.google.com), the terms “cysticercosis *Taenia solium*” were screened in the full core texts and not only in titles and keywords. Thereafter, several Google searches were performed in respect of the various countries of the region and key words and expressions such as “porcine cysticercosis”, “human cysticercosis”, “neurocysticercosis’’, “*Taenia solium*”, “*T. solium*”, and “cysticercosis” were searched in order to retrieve scientific publications on cysticercosis relating to each country. Additionally, some other internet websites, such as The Journal of Infection in Developing Countries (https://www.jidc.org), Société de Pathologie Exotique (http://www.pathexo.fr) and African Journals Online (https://www.ajol.info) were consulted for the purpose of gathering information and data which are published in local journals only.

## Results

### Data extraction

**Figure 1.**
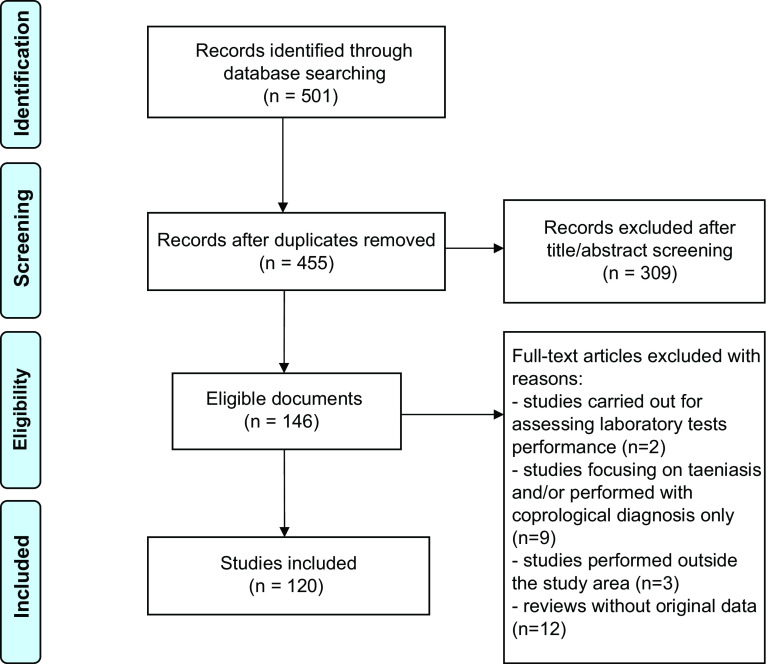
Workflow followed to identify articles in databases.

**Figure 2.**
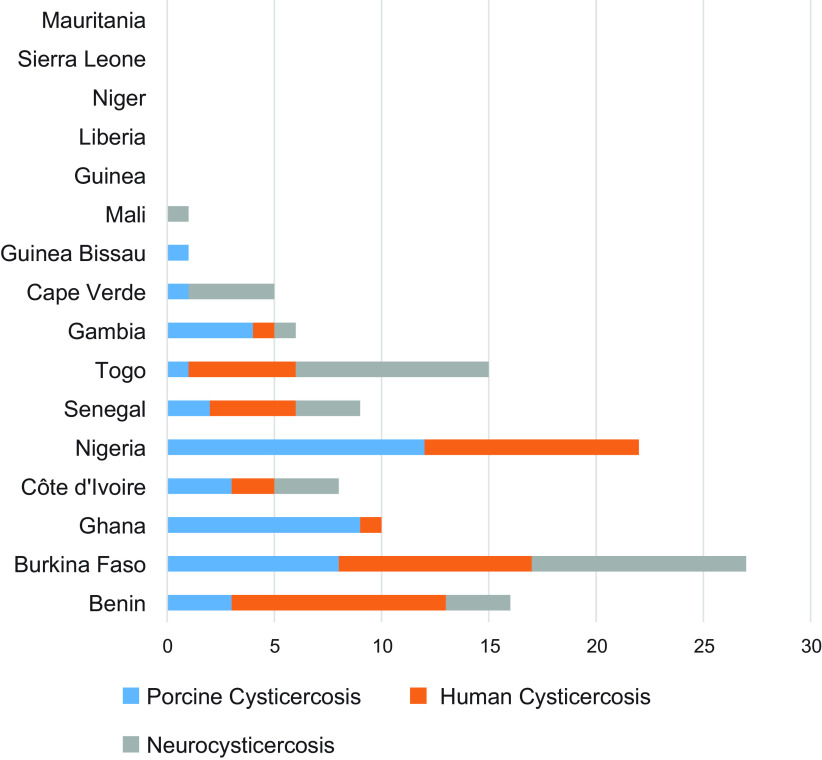
Summary of publications available by country.

Based on the online researches in databases, a total of 501 articles were identified. Redundant articles were removed (Fig. 1). All abstracts were collected, and titles and abstracts were analyzed manually. Accordingly, 146 articles were retained and full texts were obtained. Subsequently, certain articles were excluded based on the following criteria: (1) studies carried out to assess laboratory test performance (unidentified samples); (2) studies focusing on taeniasis only and/or on *T. saginata* infection; (3) studies performed outside of the study area; and (4) reviews without original data. Case reports were retained. Overall, 120 articles were included (Table 1). These articles, which cover only 11 out of the 16 countries of the region, concern the following topics: porcine cysticercosis (39), human cysticercosis (36), and neurocysticercosis (34). From these 120 articles, the following data were finally extracted and reported: country, year, prevalence, case reports and method of diagnosis (Table 2, Fig. 2).

**Table 1.** Summary of publications available (case reports and epidemiological studies).

Country name	Porcine cysticercosis	Human cysticercosis	Neurocysticercosis	No. of references (total: 120)
Benin	[60, 5, 7]	[123, 2, 64, 92, 56, 11, 55, 12, 33, 25]	[2, 13, 10]	16
Burkina Faso	[32, 53, 7, 110, 63, 73, 71, 39]	[29, 90, 18, 28, 52, 68, 18, 89, 25]	[79, 37, 87, 48, 67, 121, 119, 57, 84, 104]	27
Ghana	[8, 97, 20, 91, 7, 97, 94, 83, 10]	[21]	–	10
Côte d’Ivoire	[80, 36, 7]	[25, 58]	[13, 10, 70, 26, 86, 62]	11
Nigeria	[9, 35, 93, 61, 23, 69, 45, 46, 65, 4, 7, 75, 44]	[116, 45, 22, 81, 25, 70, 1, 75, 15, 115]	–	20
Senegal	[108, 7]	[123, 106, 56, 30]	[13, 44, 41]	9
Togo	[118]	[42, 43, 82, 56, 25]	[42, 43, 111, 16, 111, 13, 19, 118, 6]	13
The Gambia	[108, 7, 96, 98]	[103]	[107]	6
Cape Verde	[74]	–	[102, 27, 98, 95]	5
Guinea Bissau	[35]	–	[114]	2
Mali	–	–	[76]	1
Guinea	–	–	–	0
Liberia	–	–	–	0
Niger	–	–	–	0
Sierra Leone	–	–	–	0
Mauritania	–	–	–	0

**Table 2.** Epidemiological data available.

Country name	Year	Prevalence porcine cystic. (*n*)	Prevalence human cystic. (*n*)	Prevalence NCC among epileptic patients (*n*)	Methods of diagnosis	Case reports (*n*)	Ref. No.
Benin	1993	–	–	–	–	1	[11]
	1996	–	3.5 (319)	9.1 (11)	Ab-ELISA	–	[2]
	1998	–	1.3 (2625)	–	Ab-ELISA, EITB	–	[64]
	2010	0.22	–	–	Post mortem***	–	[60]
	2014	0.87 (60.924)	–	–	Tongue***	–	[59]
Burkina Faso	2000	0.57	–	–	Meat***	–	[32]
	2008	–	–	–	–	6	[18]
	2009	–	10.3–1.4–0 (532)	–	Ab-ELISA*	1	[29, 85]
	2011	32.5–39.6 (173–157)	–	–	Ag-ELISA*	–	[53]
	2012	–	–	2.2–1.5–0.2 (888)	Ag-ELISA, CT scan	–	[79]
	2012	–	4.5 (70)	–	Ag-ELISA	–	[90]
	2013	–	–	–	–	35	[104]
	2014	–	–	–	–	3**	[57]
	2016	–	0–11.5 (3609)	–	Ab and Ag-ELISA	–	[28]
Ghana	1999	11.7 (60)	–	–	Post mortem	–	[97]
	2015	2.31 (4121)	–	–	Post mortem	–	[8]
Côte d’Ivoire	1972	–	–	–	–	1	[62]
	1978	2.5	–	–	Tongue	–	[80]
	1980	–	–	–	–	1	[26]
	1991	3.6	–	–	Meat	–	[36]
	1999	–	–	–	–	4	[86]
Nigeria	1980	1.76	–	–	Post mortem	–	[35]
	1995	20.5 (2358)	–	–	Post mortem	–	[93]
	2003	–	–	–	–	1	[1]
	2010	5.85–14.4 (205)	–	–	Tongue / Post mortem	–	[61]
	2012	3.2 (247)	–	–	Post mortem	–	[23]
	2013	–	9.6 (63)	–	Ab-ELISA	1	[15, 116]
	2013	6.25 (4380)	–	–	Post mortem	1	[69, 115]
	2014	9.3 (43)	–	–	Post mortem	–	[46]
	2015	–	14.3 (300)	–	Ab-ELISA	–	[45]
Senegal	1976	–	–	–	–	2	[44]
	2010	6.4–13.2 (1705)	–	–	Ag-ELISA	–	[108]
	2011	–	11.9 (403)	23.3 (43)	Ag-ELISA, EITB, CT scan	–	[106]
Togo	1989	–	2.4 (5264)	21.6 (125)	Ag-ELISA	–	[42]
	1990	–	17 (1000)	29.5 (1000)	Ag-ELISA	–	[43]
	2000	–	38 (1000)	135.29 (1000)	Ag-ELISA	–	[16]
	2001	–	–	–	–	1	[19]
	2015	–	–	–	–	143	[82, 111]
The Gambia	2010	4.8 (371)	–	–	Ag-ELISA	–	[108]
Guinea Bissau	1980	18.4	–	–	Post mortem	–	[35]
	2015	–	–	–	–	1**	[114]
Mali	2009	–	–	–	–	1	[76]
Cape Verde	1995	–	–	–	–	1**	[104]
	2004	–	–	–	–	1**	[27]
	2013	–	–	–	–	1	[98]
	2009	–	–	–	–	1	[95]

* Ag-ELISA: Monoclonal antigen-detection enzyme-linked immunosorbent assay on serum; Ab-ELISA: Monoclonal antibody-detection enzyme-linked immunosorbent assay on serum;

** Imported in Europe;

*** Tongue or meat or post mortem inspection;

(*n*) Number of subjects or animals examined.

### Epidemiological data on porcine cysticercosis

Only 8 out of 16 countries of the region have reported porcine cysticercosis, namely Benin, Burkina Faso, Côte d’Ivoire, The Gambia, Ghana, Guinea Bissau, Nigeria, and Senegal (Table 2, Fig. 2). For some countries, data are clearly outdated, such as those for Guinea Bissau (1980), Togo (1990), and Côte d’Ivoire (1991).

Routine detection is based on tongue palpation or meat inspection, techniques characterized by low sensitivity [17, 32], which can lead to underestimation of the prevalence of cysticercosis. However, despite this low sensitivity, recent data obtained in slaughterhouses from post-mortem examinations have reported high levels of infection (from 5% to 15%), especially in Nigeria [61]. In Benin, tongue examination reported infection under 10% [59, 60]. Some data were obtained through epidemiological studies using Ag-ELISA, highlighting infection between 5% (The Gambia) and 40% (Burkina Faso) [53, 108]. In Nigeria, some chronological data are also available over the period between 1980 and 2014 [9]. During this time, prevalence increased from 1.76% in 1980 to 20.5% in 1995, fell to 3.2% in 2010 and 2012, but increased again to 9.3% in 2014. No data are available for 1991–1994, 1996 and 2009 [46]. Records were obtained through post-mortem examinations without a clear description of the protocol, with the consequence that the relevance of these fluctuations may be questioned.

In Burkina Faso, the first records (years 2000), based on meat inspection, showed a very low prevalence rate of 0.57%; thereafter, no data were provided until 2011, when, based on the Ag-ELISA diagnostic technique, seroprevalence reached 39.6% [53].

### Human cysticercosis

Seizures are the most frequent clinical manifestation of neurocysticercosis [106]. Data on neurocysticercosis are scarce and available only in terms of prevalence among epileptic patients and from the following five countries: Benin, Burkina Faso, Senegal, Nigeria and Togo (Table 2). Furthermore, Côte d’Ivoire, Mali and Cape Verde also declared case reports, though without systematic studies.

Aside from data concerning people with epilepsy, the first systematic studies on human cysticercosis were provided in 1989 and 1996 by Togo (2.4%) and Benin (3.5%), respectively. The most complete set of records relates to Burkina Faso and covers the years 2000–2016. According to the most recent data, the highest prevalence rates of human cysticercosis were found in Nigeria, with a 14.3% rate calculated on 300 persons (2015), in Senegal with an 11.9% rate calculated on 403 persons (2011), and in Burkina Faso, with an 11.5% rate calculated on 3609 persons (2016). For all these studies, serology (Ag-ELISA or Ab-ELISA, sometimes confirmed using Western Blot test-EITB) was used as the diagnosis method. Ag-ELISA was based on monoclonal antibodies that detect excretory/secretory circulating antigens of *T. solium* [16, 42, 43, 53, 79, 90, 106, 108]. Ab-ELISA was performed for the screening of serum IgG antibodies to *T. solium* by using crude antigen from cysticerci of *T. solium* [2, 28, 29, 64] or the cysticercus ELISA Kit [45, 116]. The Ag-ELISA test detects viable cysts only, that is to say, current infection caused by *T. solium* metacestodes, whereas the presence of IgG antibodies indicates exposure to *T. solium*, but not necessarily an active infection. In the same line, most of the studies among epileptic patients refer to antigen detection and rarely to antibody detection or computed tomography (CT) scans (Senegal, Burkina Faso). Data are usually old, such as in Togo (1989–1990), with a recorded prevalence rate of 29.5%. In 2011, and despite the predominance of the Muslim religion (which should involve reduced pork consumption), Senegal reported a rate of 23.3% among epileptic patients, which is remarkably high. During this period, studies in Senegal and Burkina Faso were performed in the following three phases: (1) “door-to-door” questionnaires, aimed at identifying the population at high risk for neurocysticercosis; (2) neurological examination of selected groups, aimed at confirming epileptic seizures or epilepsy in accordance with the 1989 Classification of Epilepsy Syndromes of the International League against Epilepsy [31]; and (3) serology (ELISA and EITB) and brain CT scans, to identify cysts and cysticercosis lesions.

## Discussion

### Porcine cysticercosis

In developing countries, livestock rearing is one of the main economic activities on which the poorest populations depend for food and income. Data on the prevalence of porcine *T. solium* cysticercosis are extremely scarce in West Africa and available only from a few countries. This review highlights that pork cysticercosis is largely present in most of the countries of the region. However, the burden of the animal disease (especially at local level), and its economic impact cannot be inferred from these records. While these statistics are mostly based on data provided by “official” abattoirs and slaughterhouses, it is well known that, in developing countries, most pigs are slaughtered outside these official facilities. Prevalence varies from country to country, but no data are available from Muslim countries (Niger, Mauritania, and Mali) where consumption of pork is extremely limited, with the exception of Burkina Faso, which is predominantly Muslim. However, in these countries, factors such as travel or employment of foreign housekeepers may be important sources of contamination.

Burkina Faso is estimated to have, by far, the highest prevalence of porcine cysticercosis (between 32.5% and 39.6% in 2011). Although modern pig-breeding was introduced to Burkina Faso at the beginning of the twentieth century, 80% of the pigs are still slaughtered by farmers at home and sold without prior meat inspection for cysticercosis [49], which constitutes a major risk for public health. Nigeria has the largest pig population in West Africa with 5 million animals [61]. The pig husbandry system is very similar to that used in Asia and Latin America [112], i.e. (1) intensive management, where pigs are confined within a shelter and are not allowed to move outside, (2) semi-intensive, where pigs are provided with shelter but are allowed to move outside to feed on natural vegetation, and (3) extensive or free range farming, where pigs are left to scavenge for all their food. The massive use of the free range farming system materially increases the risk of pigs being exposed to viable *T. solium* eggs and is certainly one of the factors that may have contributed to the spread of the disease in Nigeria [72]. Although a correlation between the development of *T. solium* eggs and environmental factors (e.g. rainfall, temperature and vegetation cover) has not yet been fully demonstrated, high temperature and humidity appear to have a positive impact on *T. solium* egg survival [109]. Furthermore, acidic soils in humid tropical areas may also facilitate egg survival [3, 77]. In addition, lack of regular inspections of meat (particularly in unregistered slaughter premises), despite the existence of laws and regulations, results in the consumption of unwholesome pork products [46]. In Ghana and neighboring countries (particularly Burkina Faso and Togo), the lack of cysticercosis data in the first decade of this century could be explained – at least in part – by the outbreaks of African swine fever (ASF) in 1999, which continued up to the years 2006–2007 and resulted in nearly 100% mortality in pigs [14].

These local variations appear to be largely attributable to the fact that statistics are based on the data provided by “official” abattoirs and slaughterhouses, whereas most of the pigs in endemic areas are slaughtered at home, without meat inspection. Clearly, the greatest challenge in controlling pork cysticercosis is the lack of regular reporting and data due to insufficient human resources to control slaughterhouses and herds. The organization of epidemiological surveys could thus be considered a prerequisite for monitoring the disease on a large scale. Chronological data are also needed to elaborate national strategies and launch information campaigns for rural populations.

### Human cysticercosis

According to the World Health Organization (WHO), *T. solium* causes 30% of epilepsy cases in many endemic areas of Africa, Asia and Latin America, where people and roaming pigs live in close proximity [120]. Human cysticercosis is usually found in areas where porcine cysticercosis is widespread [54, 72].

The number of publications retrieved through this systematic search was relatively limited. This may be explained by a lack of diagnostic facilities, as well as low availability of expensive neuro-imaging devices. In humans, imaging is essential to confirm the diagnosis of neurocysticercosis, based on the revised diagnostic criteria proposed by Del Brutto et al. [38]. For NCC, this scarcity of reports could be related to the condition having a status of “unrecognized” disease. Most general practitioners seem to be unaware that this disease even exists in their own countries. When recorded, official data appear not to have been collected regularly and gaps of several years between reports are frequent. Some countries (e.g. Cape Verde, Guinea and Liberia) have no official data on cysticercosis. In developing countries, case reports of neurocysticercosis used to be the only source indicating local presence of the disease. They are sometimes reported from hospitals in Europe (e.g. Italy, France, and Portugal) treating travelers coming from the region [27, 57, 102].

In Togo (1989) and Nigeria (2015), where the prevalence of human cysticercosis was 21.6% and 14.3%, respectively [42, 45], epidemiological studies have highlighted a strong association between epileptic seizures and cysticercosis. This is clearly the same situation as in Central Africa (e.g. a 2013 Cameroonian study) and in East Africa (e.g. a 2013 Rwandan study) [101], but not in The Gambia [107].

Epidemiological studies should be undertaken in all the countries of ECOWAS to fill these gaps.

## Conclusion

Concerning the scarcity of available data, *T. solium* cysticercosis remains to date a largely underestimated, if not unrecognized disease in West Africa. Importantly, in certain countries where pig production is widespread (e.g. Côte d’Ivoire or Togo), the most recent reports concerning cysticercosis date back to the early 1990s.

The major challenge for cysticercosis recognition is the lack of biological diagnosis capacities and facilities in local health institutions. Furthermore, the use of traditional pig rearing systems and the lack of adequate meat inspections, together with poor sanitation and low hygiene (e.g. defecation in open air) [123], are circumstances that contribute substantially to spread of the disease.

When future studies on cysticercosis in West Africa are carried out, three series of factors will need to be taken into consideration as they could have influenced distribution of the disease throughout the region, namely: (1) socio-demographic factors, (2) geo-historical factors, and (3) climatic and environmental factors. Epidemiological studies should be promoted in the form of health partnerships and programs implemented within the context of the ECOWAS in order to ensure that comparative results are obtained.

## Statements

### Availability of data and material

Freely available on the internet.

### Competing interests

The authors declare that they have no competing interests.

## Author contributions

JM carried out the search and wrote the text; EK and MB added information on veterinary aspects; MKS and EK added information on epidemiological aspects; RJ co-wrote the manuscript.
